# Different methods for administering 17β-estradiol to ovariectomized rats result in opposite effects on ischemic brain damage

**DOI:** 10.1186/1471-2202-11-39

**Published:** 2010-03-17

**Authors:** Jakob O Strom, Elvar Theodorsson, Lovisa Holm, Annette Theodorsson

**Affiliations:** 1 Institution of Clinical and Experimental Medicine/Department of Clinical Chemistry, Linkoping University, Linkoping, Sweden; 2 Institution of Clinical and Experimental Medicine/Department of Neurosurgery, Linkoping University, Linkoping, Sweden

## Abstract

**Background:**

Numerous stroke studies have controversially shown estrogens to be either neuroprotective or neurodamaging. The discordant results observed in rat brain ischemia models may be a consequence of discrepancies in estrogen administration modes resulting in plasma concentration profiles far from those intended. To test this hypothesis we reproduced in detail and extended an earlier study from our lab using a different mode of 17β-estradiol administration; home-made silastic capsules instead of commercial slow-release 17β-estradiol pellets. Four groups of female rats (n = 12) were ovariectomized and administered 17β-estradiol or placebo via silastic capsules. All animals underwent MCAo fourteen days after ovariectomy and were sacrificed three days later.

**Results:**

In contrast to our earlier results using the commercial pellets, the group receiving 17β-estradiol during the entire experiment had significantly smaller lesions than the group receiving placebo (mean ± SEM: 3.85 ± 0.70% versus 7.15 ± 0.27% of total slice area, respectively; p = 0.015). No significant neuroprotection was found when the 17β-estradiol was administered only during the two weeks before or the three days immediately after MCAo.

**Conclusions:**

The results indicate that different estrogen treatment regimens result in diametrically different effects on cerebral ischemia. Thus the effects of estrogens on ischemic damage seem to be concentration-related, with a biphasic, or even more complex, dose-response relation. These findings have implications for the design of animal experiments and also have a bearing on the estrogen doses used for peri-menopausal hormone replacement therapy.

## Background

During recent years substantial research efforts have been allocated to investigating the potential beneficial effects of estrogens on stroke incidence and mortality. The hypothesis that estrogens may have neuroprotective properties evolved from the observation that premenopausal women have lower risk of stroke than men of the same age have, and that stroke incidence amongst women increases in menopause [1-3]. Estrogens are widely administered as peri-menopausal hormone replacement therapy (HRT), and several large studies on human populations have been devoted to investigating the relationship between HRT and stroke. Of these, some have shown neuroprotection [4,5], while others have cast this effect of estrogens and HRT in doubt [6-9]. The largest, the Women's Health Initiative (WHI), including more than 16,000 women, was interrupted prematurely because of findings of an increased risk of coronary heart disease, breast cancer and stroke [9].

The fact that the WHI study was discontinued because of HRT-related health risks strongly reduces the possibility of performing further similar studies in human populations concerning HRT and stroke. This currently leaves us with the use of animal models to elucidate the effects of estrogens in stroke. Therefore the importance of animal studies has increased, and even more since they are sine qua non for investigating the detailed biological mechanisms of estrogens. Several studies have been performed on the question of estrogens and ischemic brain damage in animal models, rat being most commonly used. As in the human population studies the results of animal studies have been varying. Although a majority of studies have shown neuroprotective effects of estrogens [10-15], some have also shown increased damage after stroke in estrogen-supplemented animals [16-20], among these a study from our laboratory [21]. In a recent review we hypothesized that the divergent results were a consequence of differences in doses of estrogens delivered by different administration modes, commercial pellets being the only method resulting in increased lesions [22]. To test this hypothesis we designed an experiment that exactly mimicked an earlier experiment from our lab, except for the mode of administrating 17β-estradiol, the most potent of naturally occurring estrogens [21]. Silastic capsules filled with 17β-estradiol in oil were chosen since they have been shown to produce the most physiologically relevant 17β-estradiol serum levels, in contrast to the earlier used slow-release pellets from Innovative Research of America (IRA), which have been shown to produce early, very high hormone peaks [23,24]. However, the slow-release pellets are available for several doses and release-periods, and our conclusions are obviously limited to the pellets tested. Also, although the 17β-estradiol levels produced by the silastic capsules can be seen as more physiological, even this hormone profile is artificial because of its lack of natural cyclic variation. Having earlier shown neurodamage in altogether 22 rats receiving supraphysiological doses of 17β-estradiol [21], we considered it more innovative to study *when *17β-estradiol has its potential effect on cerebral ischemia by adding two groups receiving the hormone only before or only after the MCAo, respectively, rather than to include high-dose estradiol groups by means of slow release pellets or silastic capsules.

## Methods

### Animals

Forty-eight female Sprague Dawley rats were obtained from B&K Universal (Sollentuna, Sweden). The rats were kept at constant room temperature (21°C) with 12-h light/dark and sound (soft radio music) cycles for at least one week prior to the experiment. At the start of the experiment the rats were 12-14 weeks of age and weighed 270 ± 3 g (mean ± SEM). The animals were housed 2-4 in each cage, with food (Lactamin, Vadstena, Sweden) and water provided ad libitum. The cages were 20 cm high and had a floor area of 59.5 × 38.0 cm. All procedures were conducted in accordance with the National Committee for Animal Research in Sweden and Principles of Laboratory Animal Care (NIH publication no. 86-23, revised 1985). The study was approved by the Local Ethics Committee for Animal Care and Use at Linköping University. The animals used in this experiment were also included in a study of neuropeptide levels in the brain.

### Ovariectomy and 17β-estradiol administration

#### Experimental groups

**Figure 1:**
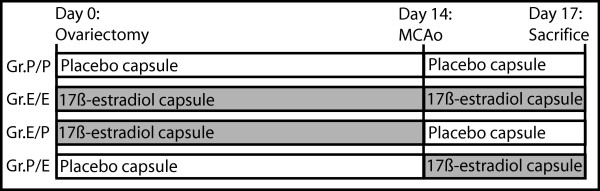
**Study flow-chart**. All animals were ovariectomized day 0 of the experiment, and at the same time Gr.P/P and Gr.P/E received placebo implants, while Gr.E/E and Gr.E/P received estrogen implants. Day 14 of the experiment, all animals underwent 60 minutes of MCAo. During the anesthesia, blood samples were drawn and the implants were replaced with new ones. Gr.P/P and Gr.E/P received placebo implants, while Gr.E/E and Gr.P/E received estrogen implants. On day 17 of the experiment, all animals were sacrificed, trunk blood collected and brains harvested for analysis.

Before experimental procedures, the rats were randomly allocated into four groups (n = 12 per group). All animals were anesthetized with 1.2-1.4% (4.2% for induction) isoflurane (Forene, Abbott, Scandinavia AB, Kista, Sweden) in an oxygen/nitrous oxide mixture (30%/70%) and ovariectomized via the dorsal route on day 0 of the experiment. One group of rats (Gr.P/P) concurrently received placebo capsules, which were substituted with new placebo capsules at the time of middle cerebral artery occlusion (MCAo) on day 14. A second group (Gr.E/E) received 17β-estradiol capsules on day 0 and new 17β-estradiol capsules on day 14. A third group (Gr.E/P) received 17β-estradiol capsules on day 0, which were substituted with placebo capsules on day 14. The last group (Gr.P/E) received placebo capsules on day 0, which were substituted with 17β-estradiol capsules on day 14 (Figure 1).

The wash-out period was omitted in the current experiment because it has been shown to have no effect on the levels of estrogens induced by the administration regimen [23].

#### Production of silastic capsules

**Figure 2:**
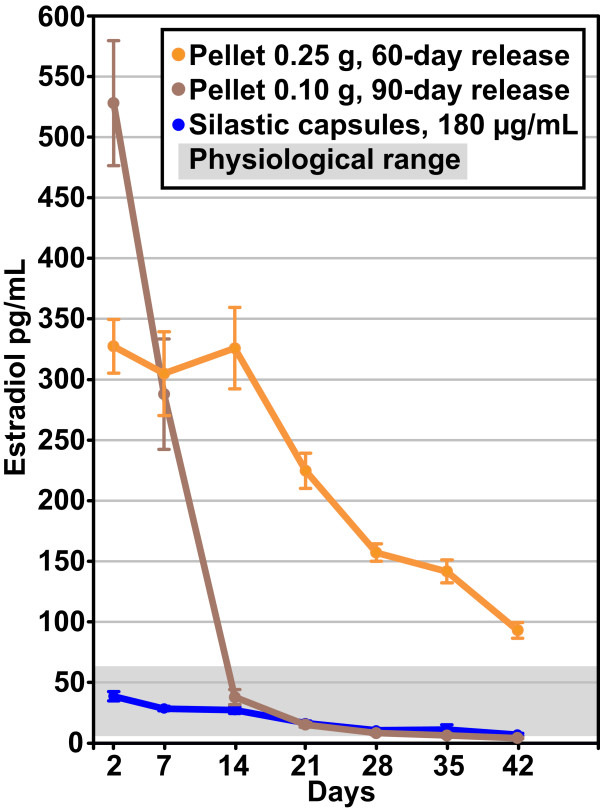
**Serum estradiol concentrations depends on administration mode**. The three most commonly-used methods for administrating 17β-estradiol to rats were tested in a previous study [23]: slow-release pellets (0.25 mg 60 day-release pellets, 0.10 mg 90 day-release pellets) and silastic capsules (silastic laboratory tubing, inner/outer diameter: 1.575/3.175 mm, filled with 20 mm columns of 180 μg 17β-estradiol/mL sesame oil). Blood samples were obtained at days 2, 7, 14, 21, 28, 35 and 42 after administration, and hormone levels were analyzed. As can be observed in the graph, silastic capsules produced 17β-estradiol concentrations that were within the physiological range for at least 4 weeks, while the pellets resulted in early supraphysiological peaks followed by a substantial decrease.

The silastic capsules consisted of thirty mm segments of silastic laboratory tubing (inner/outer diameter: 1.575/3.175 mm, Dow Corning, VWR International, Buffalo Grove, IL, USA) filled with sesame oil (placebo) or a solution of 180 μg 17β-estradiol/mL sesame oil. Five mm pieces of wooden applicator sticks (birch, length: 15 cm, diameter: 2 mm, SelefaTrade AB, Spånga, Sweden) were cut using a fine tooth saw and used to seal the silastic tubing, resulting in an oil-17β-estradiol-filled column 20 mm in length. The capsules were stored overnight in a vial containing sesame oil with the same concentration of 17β-estradiol as inside the capsules. Before implantation the capsules were carefully wiped. A 0.5 cm incision was made in the loose skin of the rat's neck, and a pocket was bluntly dissected caudally, in which the silastic capsule was gently installed using forceps. The incision was subsequently closed by a suture. This 17β-estradiol administration regimen produces hormone serum levels that are within the physiological range and significantly different to ovariectomized controls for at least four weeks (Figure 2) [23].

### Middle cerebral artery occlusion

Fourteen days after ovariectomy and silastic capsule implantation all animals underwent MCAo according to the following procedure. Anesthesia was induced by 4.2% isoflurane in a mixture (30%/70%) of oxygen/nitrous oxide in an induction chamber. A soft endotracheal tube was used for controlled ventilation (Zoovent, CWC600AP, ULV Ltd., UK) using 1.2-1.4% isoflurane in a mixture (30%/70%) of oxygen/nitrous oxide. The tidal volume and ventilation frequency were carefully regulated using on site monitoring of blood gases and acid/base status (AVL, OPTI 1 Medical Nordic AB, Stockholm, Sweden). The rats were placed with their left side up on a thermostatic heating pad (Harvard Homeothermic Blanket System, Edenbridge, UK) to maintain the core/rectum temperature at 37.0 ± 0.5°C. The left femoral artery was cannulated using a soft catheter (Micro-renathane^® ^tubing, MRE-025 Braintree Scientific Inc., MA) primed with saline containing heparin (100 IU/mL, Lovens, Malmö, Sweden) for registration of blood pressure (Blood Pressure Transducer, 56360, Stoelting, IL, USA) and pulse (Acq- Knowledge software, BioPac system, Goleta, California, USA). Using an operating microscope (Zeiss Opmi 6-H West Germany), the left MCA was exposed transcranially [25] removing part of the zygomatic bone but keeping the temporal muscle and the facial and mandibular nerves. The MCA was temporarily occluded during 60 min with a microclip between the rhinocortical branch and the lenticulostriate artery [26].

### Measuring the size of the ischemic brain lesions

On day 17 of the experiment, three days after MCAo, the rats were anesthetized by pure carbon dioxide and sacrificed by means of a rat guillotine. The brain was carefully dissected out and cooled in +4°C saline. Two millimeter thick coronary slices of the brain were cut out with razor blades directed by a rat brain matrix (RBM-4000, ASI Instrument Inc., USA) at bregma and 2, 4, 6, 8 mm posterior and 2 and 4 mm anterior to bregma. The slices were freed from the dura mater and soaked for 10 min in a solution of 2% 2,3,5-triphenyltetrazolium hydrochloride (TTC) in 0.1 mol/L PBS (pH 7.4) in a small Petri dish, maintained at 37°C in a heater. Gentle stirring of the slices was used to ensure even exposure of the surfaces to staining. Excess TTC was then drained, and the slices were scanned (ScanJet 2c, Hewlett-Packard). The size of brain lesion was measured using SigmaScan Pro version 5 (SPSS Inc.) using an automatic threshold of 40% in the green spectrum in a similar manner as described by Bederson et al. and Goldlust et al. [27,28].

### Blood samples

Blood samples for 17β-estradiol analysis was obtained by venipuncture of the hind limb into serum tubes (Vacuette^® ^Serum Tubes, Hettich Labinstrument AB, Sollentuna, Sweden) during MCAo anesthesia in all animals day 14 of the experiment. Also, five of the animals in Gr.E/E and eight of the animals in Gr.E/P had blood samples taken day 16 of the experiment. Only 700 μL of blood was withdrawn to minimize animal stress. Day 17 of the experiment, animals were sacrificed and trunk blood collected from all groups for 17β-estradiol analysis.

### 17β-estradiol radioimmunoassay

Serum levels of 17β-estradiol were analyzed using an ^125^I radioimmunoassay kit (17β-estradiol double antibody, KE2D, Siemens Healthcare Diagnostics Inc., Tarrytown, NY, USA), which has previously been tested in rat sera against other radioimmunoassays [29], and measured for 300 seconds in a gamma counter (Gamma Master 1277; Wallac-Pharmacia, Turku, Finland). This method has a lowest detectable concentration of 1.4 pg/mL and intra- and interassay coefficients of variation of 4-13% and 3.5-5.5%, respectively, depending on the concentration. All samples in the study were analyzed in one single run. Two standard curves were analyzed and all samples were read against their closest standard curve. A volume of 100 μL of serum was used for each tube in standard curves and samples. Standards and samples were analyzed in duplicate.

Four samples from a previous experiment including cycling female rats [23] were analyzed in the same batch to allow standardizing factorization of the values.

### Protocol violations

#### Excluded animals

One rat in Gr.E/P was excluded because of technical problems during surgery.

#### Excluded test results

All scanned brain slice image from two animals, one each in Gr.P/E and Gr.E/P, and one scanned brain slice image from one animal in Gr.E/E was lost in the analysis process.

Measured concentrations of 17β-estradiol in two blood samples from Gr.P/E and one blood sample from Gr.E/E were excluded due to technical problems.

### Statistics

The mean and standard error of the mean are used to describe central tendency and variation throughout the article. Size of ischemic brain lesions and 17β-estradiol concentrations were analyzed with Kruskal-Wallis nonparametric one-way analysis of variance and multiple comparisons [30]. Differences in ischemic lesion sizes between rats with and without the extra sampling occasion in Gr.E/E and Gr.E/P were analyzed by means of Mann-Whitey's test, using the same computer program as above. Differences with p-values < 0.05 were considered significant. The ischemic lesions are presented as percent of the total slice area.

## Results

### Impact of 17β-estradiol on size of ischemic brain lesion

**Figure 3:**
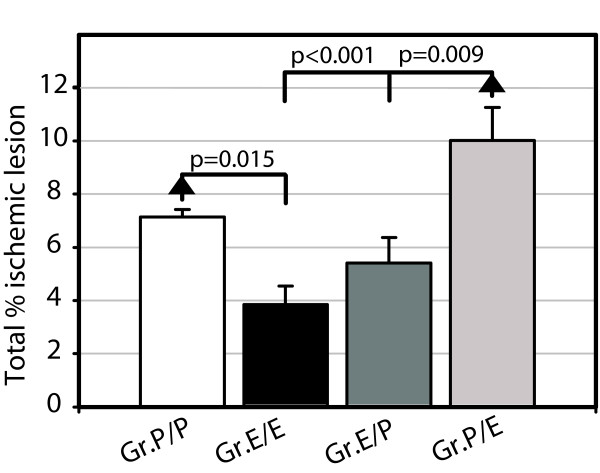
**Total ischemic brain lesion**. The mean ischemic lesion area calculated from all slices in each experimental group. Gr.P/P had significantly larger lesions than Gr.E/E (p = 0.015).

The overall mean ischemic lesion of all slices were significantly smaller in Gr.E/E (3.85 ± 0.70%) than in Gr.P/P (7.15 ± 0.27%; p = 0.015). There were no significant differences between Gr.P/P and the two groups Gr.E/P and Gr.P/E, neither in combined mean ischemic lesion area nor in any specific slice. The lesions in Gr.P/E (10.02 ± 1.23%) were significantly larger than in Gr.E/E and Gr.E/P (5.41 ± 0.95%; p < 0.001 and p = 0.009 respectively; Figure 3).

**Figure 4:**
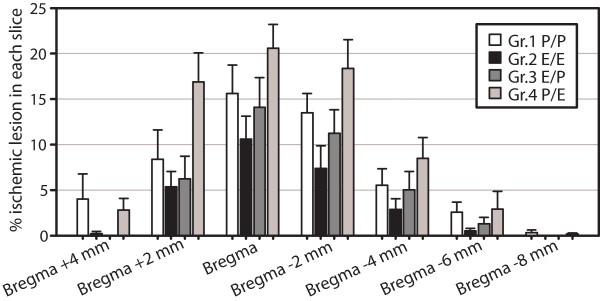
**Ischemic lesion in individual slices**. Mean areas of ischemic lesions in individual slices are presented. The lesions were largest at bregma and smallest in bregma -8 mm in all experimental groups.

The mean lesion sizes were largest in the bregma position (10.61 ± 2.49% to 20.58 ± 2.61%) and smallest in bregma -8 mm position (0.0 to 0.32 ± 0.30%) in all groups (Figure 4).

### Physiologic parameters

The blood gases and blood pressures presented in Table 1 were registered immediately prior to the MCAo.

**Table 1:** Physiological parameters in rats subjected to transient MCA occlusion

Group	n	p0_2 _(kPa)	pCO_2 _(kPa)	pH	Systolic blood pressure (mm Hg)
Gr.P/P	12	12.1 ± 0.9	5.1 ± 0.3	7.52 ± 0.04	100 ± 4

Gr.E/E	12	14.7 ± 1.3	5.2 ± 0.2	7.49 ± 0.01	113 ± 3

Gr.E/P	11	16.1 ± 1.3	5.0 ± 0.1	7.48 ± 0.01	110 ± 3

Gr.P/E	12	12.8 ± 2.4	5.5 ± 0.4	7.49 ± 0.03	105 ± 9

### Serum levels of 17β-estradiol

On the day for MCAo (day 14) Gr.E/E and Gr.E/P had significantly higher concentrations of serum 17β-estradiol than had Gr.P/P and Gr.P/E, as expected from the preceding hormone treatments (all p-values < 0.05). Day 17, the last day of the experiment, the 17β-estradiol concentrations in Gr.E/E were significantly higher than in Gr.P/P (p < 0.0001) and in Gr.E/P (p = 0.024), and the levels in Gr.P/E were significantly higher than in Gr.P/P (p = 0.0039), as expected from the second round of hormone treatments. However, the concentrations in Gr.P/E were not significantly higher than in Gr.E/P. Serum 17β-estradiol concentrations are presented in Table 2.

**Table 2:** Concentrations of 17β-estradiol (mean ± SEM pg/mL)

Group	Day 14 (MCAo)	Day 16	Day 17 (sacrifice)
Gr.P/P	1.5 ± 0.7	Not measured	2.5 ± 1.8

Gr.E/E	10.1 ± 5.4	35.6 ± 8.5	32.4 ± 8.5

Gr.E/P	12.8 ± 4.7	8.0 ± 4.6	7.7 ± 2.1

Gr.P/E	1.6 ± 1.0	Not measured	11.8 ± 3.5

### Possible effect of venipuncture on the size of the ischemic lesion

The mean lesion area of the thirteen rats in Gr.E/E and Gr.E/P that underwent an extra venipuncture on day 16 did not differ from the other rats in these groups (4.71 ± 0.57% versus 4.34 ± 0.50%; p = 0.38).

## Discussion

17β-estradiol protects against ischemic brain damage from transient MCAo in female Sprague Dawley rats when administered to provide physiological serum concentrations using silastic capsules both before and after MCAo. No significant neuroprotection was found when the 17β-estradiol was administered only during the two weeks before or the three days immediately after MCAo.

The main purpose of the current experiment was complementing an earlier study from our lab, using a different method for 17β-estradiol administration, and administering 17β-estradiol only before or after the ischemic episode. Silastic capsules were used in the present study instead of slow-release pellets from the company IRA in the previous study (Table 3) [21]. Since neuroprotection of 17β-estradiol was found in the current study, while the opposite was found in the previous experiment, the choice of administration method seems to have a crucial effect on the results. Importantly, this effect is not confined to producing different degrees of neuroprotection, but the slow-release pellets actually cause neurodamage instead of neuroprotection. This is in concordance with a previous review comparing methods of studies reporting neuroprotection versus neurodamage of estrogen, showing that 17β-estradiol has only been reported to be neurodamaging when slow-release pellets from IRA have been used [22].

**Table 3:** Methodological comparison of previous and present study

Methodological parameter	Theodorsson & Theodorsson 2005	Present study
Estrogen administration mode	Pellets from Innovative Research of America	Home-made silastic capsules

Length of estrogen treatment	2 weeks	

Type of estrogen	17β-estradiol	

Method for induction of ischemic lesions	MCAo by microclip, reperfusion after 60 min	

Length of time between ischemia and damage evaluation	72 hours	

Type of outcome measure	TTC-staining and infarct size measurement	

Rat sex	Female	

Rat strain	Sprague-Dawley	

Rat age	3 months	

Rat diseases	None	

Miscellaneous	Same laboratory, surgeon and technical equipment used in both studies	

**Effect of 17β-estradiol on cerebral ischemia**	**Neurodamage (p < 0.001)**	**Neuroprotection (p = 0.015)**

The reasons for discrepancies in results from the silastic capsule and pellet method of administration of 17β-estradiol are probably the markedly different serum concentration profiles produced (Figure 2). In previous experiments in our lab we have shown that the pellets result in extreme, supraphysiological levels for several days or weeks, followed by a substantial decrease, while silastic capsules produces serum levels that are, although steadily decreasing, within the physiological range for at least four weeks [23,31]. In the light of these administration method studies it seems reasonable to conclude that the early, prolonged, supraphysiological peak of the pellets in the context of cerebral ischemia exerts detrimental effects on the brain.

The time aspect of estrogens' effects on cerebral ischemia, investigated in the current study, has been addressed in a few previous studies. In 1997, Simpkins et al. compared infarct volumes when 17β-estradiol had been intravenously administered 24 h before, 40 min and 90 min after ischemic damage respectively and found a protective effect at all three time points [10]. In 2000, Yang et al. administered 17β-estradiol through intravenous injections and silastic capsules (filled with crystalloid hormone, differing from the oil-solution adopted in the current experiment) at 0.5, 1, 2, 3 and 4 hours after onset of ischemia finding less neuroprotection with later administration [32]. Saleh et al. conducted two experiments in 2001 where the time aspect of estrogens effect on cerebral ischemia was investigated. In one, subcutaneous injections of 17β-estradiol were administered at 30 min before ischemia, at the onset of ischemia and at 30 min after the onset of ischemia respectively. It was found that administration 30 min before ischemia produced the most substantial neuroprotection, and that administration 30 min after ischemia did not significantly influence the infarct size [12]. In the other, the hormone was administered by intraventricular infusion at 30 and 10 min before ischemia respectively, with the result that more protection was obtained from the earlier time point [33]. In 2007, Liu et al. administered subcutaneous injections of 17β-estradiol at 30 min and at 6 hours after onset of ischemia respectively, only finding neuroprotection from the first time of injection if all rats were counted [34]. The narrow time span for hormone administration, differing at most 25.5 h [10], and the administration methods adopted in all of these previous studies contrast strongly with the current study, which makes precise comparisons difficult. However, our present observations suggest that administering estrogens by means of slow-release silastic tubes at the time of ischemia lacks the protective properties of a long-term estrogen treatment, which is in contrasts to earlier results [10,32,34]. Thus, the current results indicate that administering 17β-estradiol only at the time of stroke by silastic capsules is ineffective, and that the protective potential of 17β-estradiol is facilitated by prophylactic long-term administration. However, the impact of estrogens during the development of the ischemic damage is suggested by the fact that no significant protection was observed in Gr.E/P, receiving only estrogen pretreatment, while protection is seen when at-stroke estrogens are added to this regime, as in Gr.E/E.

Similar reasoning can be applied when considering the results from Gr.E/P. Although this regime seems to lack protective properties by itself, the partial neuroprotective of estrogen pretreatment is suggested by the lack of protection in Gr.P/E in comparison to Gr.E/E. To our knowledge, no study investigating the effect of 17β-estradiol pretreatment, interrupted at the time of ischemic damage, has been previously reported.

The 17β-estradiol concentrations in Gr.E/P and Gr.P/E did not differ significantly on the last day of the experiment. Because the half-life of 17β-estradiol in the blood is measured in minutes, the comparatively small decrease in hormone levels in Gr.E/P was unexpected. A possible reason could be that although the hormone-containing capsules had been replaced with placebo capsules, some of the hormone could still have been left in the subcutis. This hypothesis is supported by the lack of hormone level decrease between days 14 to 17 in Gr.E/P.

### Possible mechanisms

The elucidation of the mechanisms behind estrogens effects on cerebral ischemia is of fundamental importance for the possibility of directly affecting the pathological process. Many mechanisms have been suggested to be involved in the protective effects of 17β-estradiol in the brain, being mediated by intracellular receptors, membrane receptors and by direct chemical interactions including antioxidant effects [35]. Interestingly, the antioxidant effects of estrogens (phenolic scavenging of lipid peroxyl radicals) may prevail at physiological concentrations whereas redox-cycling at higher estrogen concentrations may lead to increased radical generation and damage [36,37]. Estrogens may also protect against neurodamage by antithrombogenic actions through effects on smooth vascular muscle and endothelium, systemic effects reducing total cholesterol and LDL concentrations, and through effects on hepatic expression of genes for several coagulation and fibrinolytic proteins. Also, estrogens have direct effects on neurons by increasing transcription of antiapoptotic genes, decreasing transcription of apoptotic genes, reducing mitochondrial release of Cytochrome C, interacting with neurotrophins, and have anti-inflammatory effects through attenuation of TNF-α (reviewed in [38,39]). Damaging effects on the nervous system by estrogens have been suggested to result from the formation of free radicals [40] or from increased excitotoxicity [41] by up-regulating glutamate NMDA receptors [42], inducing seizure activity [43] and decreasing glutamate uptake by astrocytes [44]. Thus, it seems reasonable that all these mechanisms, differing in both route of exertion and individual dose-response relations, together could create a highly complex, e.g. biphasic, dose-response relation.

## Conclusions

The hypothesis that the mode of estrogen administration dictates the hormone's effect on ischemic damage by producing specific hormone release profiles is corroborated by the present study. It is important to realize that this is not merely a methodological question concerning rat models, but suggests a fundamental property of estrogens' actions in the nervous system. Thus, it does not only have relevance for the design of future animal studies; it also has a bearing on the debate on peri-menopausal hormone replacement therapy. Perhaps altered administration regimens resulting in lower plasma concentrations may reduce the hazardous effects and even have favorable effects, while preserving the relief of peri-menopausal symptoms?

## Abbreviations

Gr.E/E: Experimental group receiving 17β-estradiol capsules both before and after MCAo; Gr.E/P: Experimental group receiving17β-estradiol capsules before and placebo capsules after MCAo; Gr.P/E: Experimental group receiving placebo capsules before and 17β-estradiol capsules after MCAo; Gr.P/P: Experimental group receiving placebo capsules both before and after MCAo; IRA: Innovative Research of America; MCA: Middle cerebral artery; MCAo: Middle cerebral artery occlusion; TTC: 2,3,5-triphenyltetrazolium hydrochloride.

## Competing interests

The authors declare that they have no competing interests.

## Authors' contributions

JOS contributed by participating in planning/designing the study, producing the 17β-estradiol capsules, performing the hormone assays, assessing the data, and by drafting the article. ET contributed by participating in planning/designing the study, assessing the data and by revising the manuscript. LH contributed by performing all surgical procedures, obtaining all necessary samples, analyzing infarct sizes and by revising the manuscript. AT contributed by participating in planning/designing the study, analyzing infarct sizes, assessing the data and by revising the manuscript. All authors read and approved the final manuscript.
